# Dynamic resource allocation drives growth under nitrogen starvation in eukaryotes

**DOI:** 10.1038/s41540-020-0135-y

**Published:** 2020-05-15

**Authors:** Juan D. Tibocha-Bonilla, Manish Kumar, Anne Richelle, Rubén D. Godoy-Silva, Karsten Zengler, Cristal Zuñiga

**Affiliations:** 1 Bioinformatics and Systems Biology Graduate Program, University of California, San Diego, 9500 Gilman Drive, La Jolla, CA 92093-0760 USA; 2 Department of Pediatrics, University of California, San Diego, 9500 Gilman Drive, La Jolla, CA 92093-0760 USA; 3 Grupo de Investigación en Procesos Químicos y Bioquímicos, Departamento de Ingeniería Química y Ambiental, Universidad Nacional de Colombia, Bogotá, DC Colombia; 4 Department of Bioengineering, University of California, San Diego, La Jolla, CA 92093-0412 USA; 5 Center for Microbiome Innovation, University of California, San Diego, 9500 Gilman Drive, La Jolla, CA 92093-0403 USA

**Keywords:** Biochemical networks, Dynamical systems

## Abstract

Cells can sense changes in their extracellular environment and subsequently adapt their biomass composition. Nutrient abundance defines the capability of the cell to produce biomass components. Under nutrient-limited conditions, resource allocation dramatically shifts to carbon-rich molecules. Here, we used dynamic biomass composition data to predict changes in growth and reaction flux distributions using the available genome-scale metabolic models of five eukaryotic organisms (three heterotrophs and two phototrophs). We identified temporal profiles of metabolic fluxes that indicate long-term trends in pathway and organelle function in response to nitrogen depletion. Surprisingly, our calculations of model sensitivity and biosynthetic cost showed that free energy of biomass metabolites is the main driver of biosynthetic cost and not molecular weight, thus explaining the high costs of arginine and histidine. We demonstrated how metabolic models can accurately predict the complexity of interwoven mechanisms in response to stress over the course of growth.

## Introduction

Perturbations in environmental conditions require organisms to withstand transient phases from nutrient abundance to nutrient depletion. Adequate physical and metabolic responses enable microorganisms to survive under these dynamic environments^1^. As the three main requirements for growth, the principal drivers of metabolic shifts are carbon, nitrogen, and energy sources. Several studies have focused on identifying the response mechanism of organisms to the depletion of each of the sources^2^. Metabolic adaptation has been associated with changes in the production or degradation rates of biomolecules. These profiles are observed in various heterotrophic and photoautotrophic microorganisms, increasing their resistance to stress conditions and nutrients limitation^1,3–12^. Depletion of carbon and energy sources directly reduce the synthesis of all biomass precursors since carbon is the backbone of nucleic acids, proteins, lipids, and carbohydrates and all anabolic pathways consume energy. On the other hand, nitrogen depletion has been proven to selectively decrease the synthesis of proteins and nucleic acids, triggering a metabolic response that upregulates the synthesis of carbon-rich compounds^13^. Previous studies on the metabolic response mechanism to nitrogen depletion have identified key signal metabolites^13^ and global biomass composition trends^14,15^. However, an in-depth analysis at the genome scale has not yet been employed to understand how metabolic pathway use changes to survive in nutrient-deplete conditions.

M-model simulations allow to identify main metabolic reactions driving growth phenotypes under diverse genetic and environmental conditions^15,16^. Using a reduced number of known uptake rates, or constraints^17^, M-model simulations predict growth and flux distributions (phenotypes) under diverse genetic and environmental conditions, identifying the main drivers of metabolism^14^. In this approach, the formulation of the biomass objective function (BOF) is highly important to obtain biologically relevant flux distributions. Each biomass precursor in the BOF pulls resources from the network depending on its stoichiometric coefficient; ideally each coefficient is experimentally determined. Considering this dependence, we devised a strategy to compute dynamic flux distributions by using time-course biomass composition data, thus expanding the scope of M-models from a steady state to several pseudo-steady states encompassing growth under stress conditions and in time-dependent processes^1^.

We employed this dynamic simulation procedure to study the metabolic effect of nitrogen depletion, a common and important phenomenon in nature and various biotechnology processes^15,18^. We used time-course composition data to define BOFs at six timepoints of culture for five eukaryotic microorganisms (phototrophs *Chlorella vulgaris* and *Phaeodactylum tricornutum* and heterotrophs Chinese Hamster Ovary cells, *Saccharomyces cerevisiae*, and *Yarrowia lipolytica*). High quality and validated M-models exist for all five organisms^3,19–22^. Then, flux balance analysis (FBA) using the COBRA Toolbox^23^ was employed to calculate flux distributions at each timepoint and growth mode (heterotrophic or photoautotrophic). We predicted variations in flux distributions that reveal time-specific metabolic activities across compartments, thus describing the metabolic response of different organisms to nitrogen depletion. We further explain how flux distributions are shaped by changes in the biomass composition by calculating the component-specific impact on growth simulations, which is referred to in this work as sensitivity. The sensitivity of a component was calculated as the variation of growth rate due to a change in its abundance in the biomass. Our results show that sensitivity holds a tight relationship with the biosynthetic cost of each component, which placed lipids of high molecular weight as the most impactful on growth. However, we showed through a deeper look into the biosynthetic cost of amino acids that molecular weight does not entirely explain the cost of biomass components, as was the case for arginine and histidine. Rather, we found that the underlying driver of cost is the free energy contribution of the chemical groups contained in the biomass components.

## Results

### Dynamic metabolism associated with nutrients depletion can be simulated using metabolomics data

**Fig. 1:**
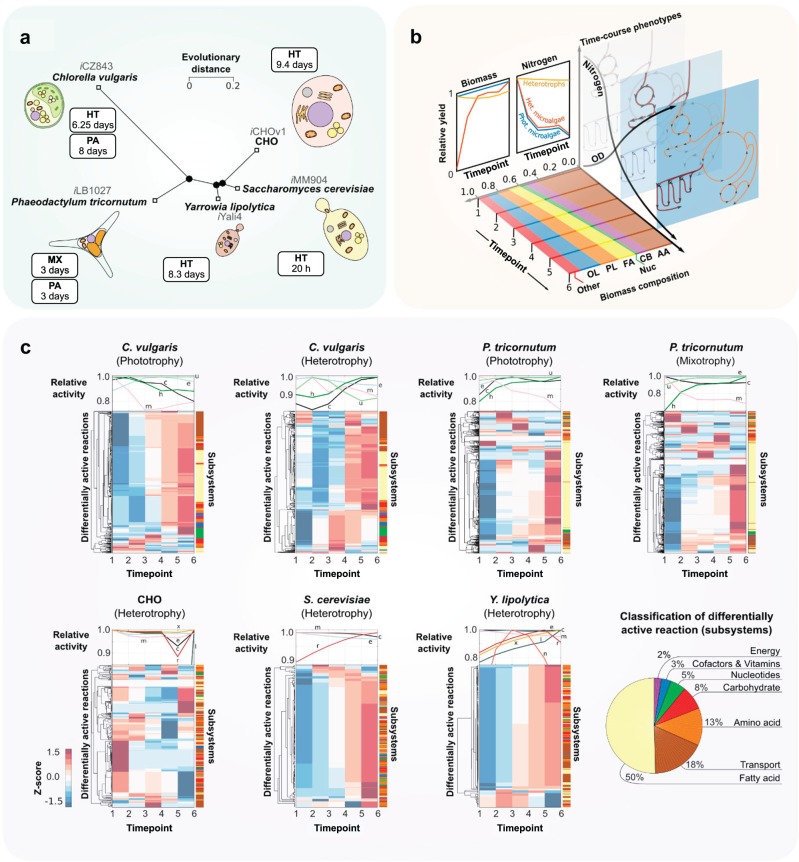
Prediction of metabolic trends using time-course metabolomics data of eukaryotic organisms. **a** Neighbor-joining tree based on almost full-length RimM rRNA gene sequences, showing phylogenetic relationships between the phototrophs *C. vulgaris*, *i*CZ843^3^, and *P. tricornutum*, *i*LB1027^24^, and the heterotrophic cell factories Chinese Hamster Ovary cell, *i*CHOv1^19^, *S. cerevisiae*, *i*MM904^22^, and *Y. lipolytica*, *i*Yali4^20^. Timespans of culture for each organism are shown in the boxes for heterotrophy (HT), mixotrophy (MX), and photoautotrophy (PA). **b** Available metabolomics data were retrieved and used to compute time- and condition-specific BOFs. Biomass components are abbreviated as follows: amino acids (AA), carbohydrates (CB), nucleotides (Nuc), fatty acids (FA), phospholipids (PL), and other lipids (OL). **c** Cluster analysis of reaction flux variations. *Z*-scores were computed from the change in flux of a reaction from one timepoint to the next, or in other words, horizontally. On top of each clustergram we show time-course carbon load as a measurement of compartment activity, in order to correlate it with reaction flux variations. To permit the comparison among all organisms, relative activity was calculated as the ratio of the activity at each timepoint versus the initial activity (see activity in Methods). Compartment abbreviations are: cytosol (c), mitochondria (m), chloroplast (h), extracellular environment (e), thylakoid lumen (u), endoplasmic reticulum (r), glyoxysome (x), lysosome (l), and nucleus (n). The pie chart in the bottom right shows the classification in subsystems of all differentially active reactions across models.

M-models compile metabolic knowledge in a mathematical framework, enabling a mechanistic understanding of cell physiology based on observations (constraints). We included time-course metabolomics data in the M-models of the phototrophs *C. vulgaris*, *i*CZ843^3^, and *P. tricornutum*, *i*LB1027^24^, and the heterotrophic cell factories Chinese Hamster Ovary cell, *i*CHOv1^19^, *S. cerevisiae*, *i*MM904^22^, and *Y. lipolytica*, *i*Yali4^20^ (Fig. 1a). The general properties of the M-models are shown in Table 1.

**Table 1:** Characteristics of included genome-scale metabolic models.

#	Organism	Model ID	Mode ^a^	Genes	Reactions	Metabolomics dataset source	Culture timespan of dataset
1	*Chlorella vulgaris*	*i*CZ843^3^	P	843	2294	Zuñiga et al.^1^	8 days
2			H			Zuñiga et al.^1^	6.25 days
3	*Phaeodactylum tricornutum*	*i*LB1027^21^	P	1027	4456	Levitan et al.^4^, German-Báez et al.^8^, Parsons et al.^9^, Siron et al.^10^, Yang et al.^11^, Willis et al.^12^	3 days
4			M				
5	*Yarrowia lipolytica*	*i*Yali4^20^	H	901	1985	Rakicka et al.^7^	8.3 days
6	*Saccharomyces cerevisiae*	*i*MM904^22^	H	905	1577	Bu et al.^6^	20 h
7	CHO cell	*i*CHOv1^19^	H	1766	6663	Selvarasu et al.^5^	9.4 days

^a^Growth condition (Mode) is abbreviated as follows: photoautotrophic (P), mixotrophic (M), and heterotrophic (H) growth conditions.

Experimental biomass composition measurements, determined throughout the growth of all eukaryotic cells while facing nitrogen starvation, were used to formulate dynamic biomass reactions for all organisms. For *C. vulgaris*, data were collected under both heterotrophy and photoautotrophy, and for *P. tricornutum* under mixotrophy and photoautotrophy. All datasets were aligned in six timepoints (Fig. 1b) representing as follows: (1–2) nitrogen-replete stage, (3–4) transition stage, and (5–6) nitrogen-deplete stage (see definition of timepoints in Methods). Furthermore, we show in Supplementary Fig. 1 the outstanding difference of experimentally determined BOFs with the composition predicted by the genome, as previously suggested for bacteria^25^, which highlights the improvement by integrating metabolomics data.

Regardless of evolutionary distance, the biomass composition of all five eukaryotes showed a common response (Supplementary Fig. 1) of increasing carbon- and energy-rich components, namely acylglycerols (*R*^2^ = 0.51, *p* = 1.1 × 10^−^^6^), phospholipids (*R*^2^ = 0.58, *p* = 7.6 × 10^−8^), and other lipids (*R*^2^ = 0.84, *p* = 3.8 × 10^−10^), such as pigments, sulfolipids, and glycolipids. We used experimental measurements of components present in the BOF, predicting dynamic variations in flux distributions as well as in total organelle activity and metabolite exchange across compartments (cross-talk). In Fig. 1c, a cluster analysis (see Methods) of predicted reaction flux variations with subsystem classification is shown. We found variations in fatty acid metabolism, amino acid metabolism, and cytosol–mitochondria transport processes. In all cases, protein synthesis decreased due to low nitrogen concentrations in the culture medium, whereas carbohydrate and fatty acid metabolisms were upregulated to store carbon, as previously reported for eukaryotes^1,15^. This metabolic trade-off affects the middle growth stage of all cells, but in CHO cells takes place at the early stage (timepoints 1–2). Consequently, the nitrogen-depletion threshold that triggers lipid synthesis appears to be much more sensitive for this organism. CHO cells are not capable of synthesizing some proteinogenic amino acids such as asparagine, arginine and aspartate, among others. Hence, the depletion of carbon and nitrogen is intertwined as a result of these amino acids being provided in the medium. Our simulations of biomass yield show that non-glucose carbon input can amount to 45% of the biomass c-mol at the highest (first timepoint in Fig. 1b).

In microalgae, clusters were shown to predominantly contain reactions of the same subsystem, with a clear segregation in *C. vulgaris* for fatty acid metabolism and transport. In *P. tricornutum*, transport clustered with reactions of other subsystems, whereas fatty acid metabolism showed a separation similar to that in *C. vulgaris*. This orchestration of lipid synthesis as a response to nutrient starvation in microalgae has been widely reported^15^, and is the reason they are a focus of industrial research for biotechnological purposes. In CHO, *S. cerevisiae* and *Y. lipolytica*, subsystems did not clearly explain the clustering of reactions. Results show that fatty acid metabolism reactions were combined with clusters of amino acid metabolism and membrane transport (Fig. 1c). Opposite to the phototrophic organisms, *S. cerevisiae* and *Y. lipolytica* showed two clearly distinct clusters, displaying a more visible global trend of metabolic shift after nutrient depletion. This suggests a highly intertwined variation across the metabolism for the heterotrophs. In general, these three subsystems contained the highest number of varying reactions.

**Fig. 2:**
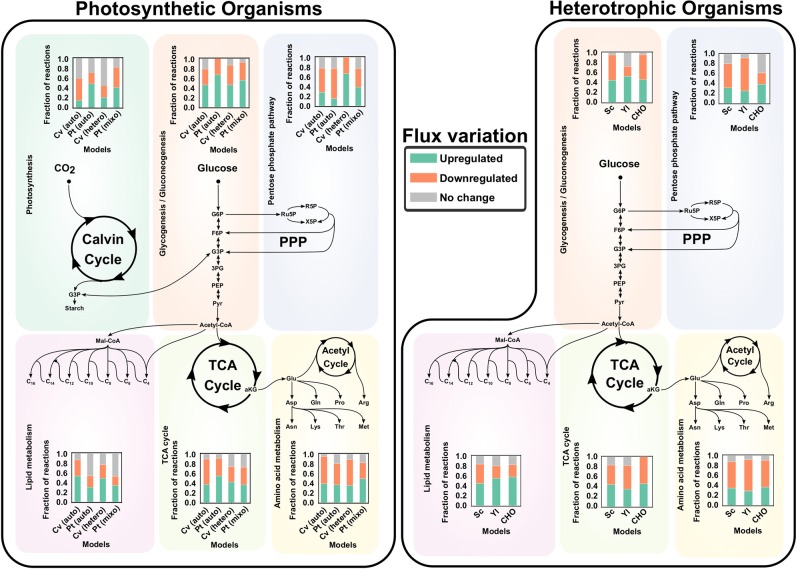
Pathway activity variation of core metabolism in photoautotrophic (left) and heterotrophic (right) organisms after nitrogen depletion. The solution space of the models was sampled according to a previously reported methodology^53^; employing functions available in The COBRA Toolbox (see Methods). Significant upregulations or downregulations was assessed by performing a Student’s *t* test on the distributions of each reaction at the initial and final time points. Included organisms are abbreviated as follows: *Chlorella vulgaris* (Cv)*, Phaeodactylum tricornutum* (Pt)*, Saccharomyces cerevisiae* (Sc), *Yarrowia lipolytica* (Yl), and Chinese Hamster Ovary cells (CHO).

Predicted flux distributions unravel changes in each organelle activity/load (see Methods for a detailed definition of organelle load). We determined carbon (Fig. 1) and nitrogen (Supplementary Fig. 4) loads over the course of growth of each model and growth condition by calculating compartment-level carbon and nitrogen transport rates. We identified a ubiquitous trend of mitochondrial carbon and nitrogen load reduction, caused by the activity reduction in reactions of energy (tricarboxylic acid cycle (TCA) and oxidative pentose phosphate pathway (PPP)) and amino acid metabolism (Fig. 2).

Moreover, a significant share of the global mitochondrial activity variation was associated with change of the activity of other organelles. The reduction of fast-growth-related fluxes induced a decrease in metabolite transport rates among the mitochondria, cytosol, and the chloroplast. This mitochondrial communication with other organelles has previously been reported to help regulate several energetic cellular functions^26^. Moreover, we identified a common decrease after nitrogen depletion in the transport of amino acids, as well as their precursors, e.g., α-ketoglutarate (AKG). In the non-steady-state of living cells, a lower transport rate of AKG would mean its accumulation in the cytosol, which has been reported to be a consequence of nitrogen depletion^13^. The direct link of AKG levels to nitrogen availability rendered it one of the principal signal metabolites of low nitrogen to carbon availability ratio^2,13^. Other amino acid precursors whose transport rates were found to decrease were succinate, fumarate, and O-acetyl carnitine (phosphoketolase pathway in *P. tricornutum*), and tetrahydrofolate (THF).

We predicted that in most cases the cytosol activities exhibited a long-term (through the timespan of culture) decrease, and at the same time amino acid biosynthetic reactions were downregulated in all organisms (Fig. 2). Despite the stress-induced reduction of energy and amino acid production, the microalgae exhibited a long-term increase in cytosol and chloroplast activity. In *P. tricornutum* and in *C. vulgaris* (during heterotrophic growth), the cytosol and chloroplast carbon and nitrogen load increased. For *C. vulgaris*, simulations show that the increase of cytosol and chloroplast activity is mainly due to highly active symport of asparagine and protons between these two compartments. Protons are transported from the chloroplast to the cytosol, where they are subsequently used for ATP regeneration in this compartment, as well as in the mitochondria.

In *P. tricornutum*, activity increase represents the fraction of upregulated reactions (Fig. 2) by photosynthetic pathways. As shown by our simulations and in a previous report using proteomics and expression data for *P. tricornutum*^27,28^, these reactions correspond to carbon fixation (included in the group of reactions labeled as photosynthesis in Fig. 2 and in Supplementary File 1). Remmers et al.^27^ reported the upregulation of protein abundance of a vast majority of carbon fixation enzymes, which supports the increased flux in our calculations. Interestingly, other regulatory changes in *P. tricornutum* highlighted by this proteomics dataset could be predicted by our simulations. Around 21% of carbohydrate metabolism and 20% of lipid metabolism proteins were observed to be upregulated, in accordance with the predicted increase in 37% and 29.9% reaction fluxes in these subsystems (Supplementary File 2), respectively, and the consequential long-term rise in the cytosol and chloroplast carbon loads.

### Identifying biomass metabolites driving growth phenotypes

A sensitivity analysis was conducted to analyze the effect of changes in stoichiometric coefficients of the BOF on predicted growth rates of all organisms. This analysis was based on the definition of sensitivity as the relationship among the change in a controlled variable (BOF-metabolite *i*) and the change of a response variable. With growth rate as the response variable, the sensitivity of metabolite *i* represents the variation of the predicted growth rate caused by a change in the composition of *i*. The sensitivity of a metabolite is not constant across metabolic networks, as different biomass compositions affect the flux distributions and responsiveness of the whole network. Therefore, we performed a codependence analysis in which hundreds of sensitivities were calculated for a single metabolite, so that the mean of the distribution could be used as a representative value of the sensitivity (see Methods for a complete definition of sensitivity and codependence).

**Fig. 3:**
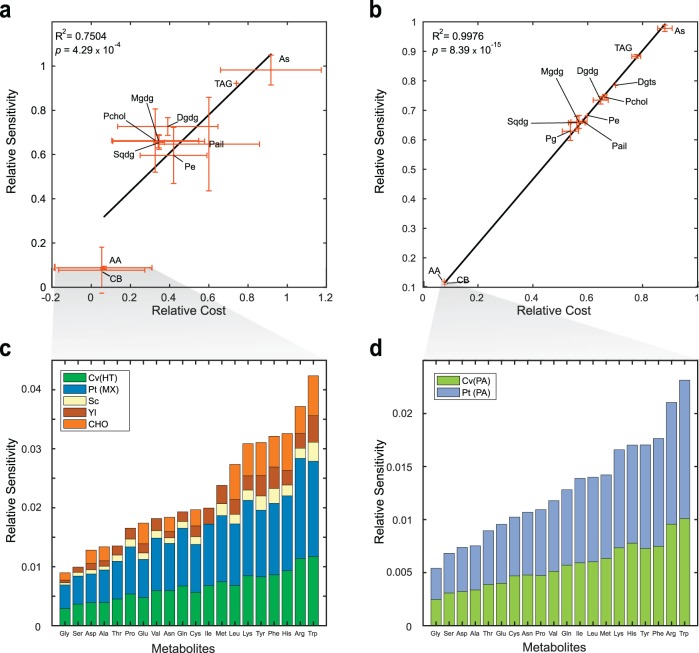
Correlation of sensitivity with biosynthetic cost for heterotrophic or mixotrophic (HT and MX, a and b) and photoautotrophic (PA, b and d) growth conditions. **a**, **b** Sensitivity values were grouped according to the classification of the biomass precursor, them being: amino acids (AA), carbohydrates (CB), triacylglycerols (TAG), all *cis*-sulfoquinovosyl diacylglycerol (As), sulfoquinovosyl diacylglycerols (Sqdg), monogalactosyl diacylglycerol (Mgdg), digalactosyl diacylglycerol (Dgdg), phosphatidylcoline (Pchol), phosphatidyl inositol (Pail), and phosphatidyl ethanolamine (Pe). As done for Fig. 1, we calculate relative activity as the ratio of the activity at each timepoint versus the initial activity to allow for comparisons across organisms (see activity in Methods). We identified the effect of molecular weight on sensitivity, as smaller molecules such as amino acids and carbohydrates with molecular weights from 137 to 238 g mol^−1^ showed sensitivities at least 4 times lower than those of lipids, whose molecular weights range from 737 to 1080 g mol^−1^. **c**, **d** Amino acids are sorted from smallest to biggest sensitivities. Included organisms are abbreviated as follows: *Chlorella vulgaris* (Cv), *Phaeodactylum tricornutum* (Pt), *Saccharomyces cerevisiae* (Sc), *Yarrowia lipolytica* (Yl), and Chinese Hamster Ovary cells (CHO).

We calculated the biosynthetic cost for every metabolite in the biomass reaction of the five models, as the amount of ATP molecules needed to produce one molecule of each biomass precursor. We then correlated the sensitivities with the calculated costs, and we found a strong correlation between them for both autotrophic (*R*^2^ = 0.998, *p* = 8.4 × 10^−15^) and heterotrophic (*R*^2^ = 0.75, *p* = 4.3 × 10^−4^) growth, meaning that regardless of growth condition and organism, sensitivity was driven by biosynthetic cost (Fig. 3). Same results were obtained for NADH and NADPH cost (see correlation coefficients in Supplementary File 3).

Modeling predictions suggest that molecular weight of the biomass precursors is one of the major drivers of their biosynthetic cost and growth (Fig. 3a, b), regardless of phylogeny and growth mode. Even though a higher diversity of heterotrophic organisms induced greater standard deviations of calculations, a remarkably similar trend was exhibited with respect to organisms growing photoautotrophically. Due to the high connectivity of amino acids we analyzed their sensitivity in Fig. 3c, d. We found that the correlation holds not only for the macroscopic trend (correlation for all biomass components in Fig. 3a, b), but also for the correlation among amino acid cost and sensitivity. Our results are in sync with previous findings in which tryptophan the biggest and most expensive amino acid^29^ exhibits the highest sensitivity, opposite to the obtained for smaller molecules such as glycine, serine, and aspartate.

### Metabolite-specific energetics shape growth phenotypes

Amino acids with higher carbon and nitrogen bounds, such as tryptophan and phenylalanine (nine carbons), can affect growth roughly four to five times more than low molecular weight amino acids such as glycine (two carbons), as shown in Fig. 3c, d. This assumption has driven the hypothesis that sensitivity and biosynthetic cost are correlated with molecular weight, as previously reported^30^. However, our sensitivity results also demonstrated that arginine and histidine (six carbons) are competitive drivers of growth, comparable to the most expensive amino acid tryptophan^30^, which has been identified as the most expensive amino acid of bacterial metabolism^31^. In previous reports, arginine and histidine exhibited unexceptional costs of merely 27 and 38% of the biosynthetic cost of tryptophan (73–75.5 mole ATP/mole Trp)^29,30,32,33^, as opposed to our results of more than 70%.

To gain insight into this finding, we evaluated the effect of simultaneous variations in nitrogen content and molecular size on biosynthetic cost. Supplementary Fig. 6 shows the interaction among N/C ratio and cost. As expected, higher carbon content induces higher biosynthetic cost than nitrogen for most compounds; however, for arginine and histidine (the most nitrogen-rich compounds), nitrogen content was the major driver of cost.

**Fig. 4:**
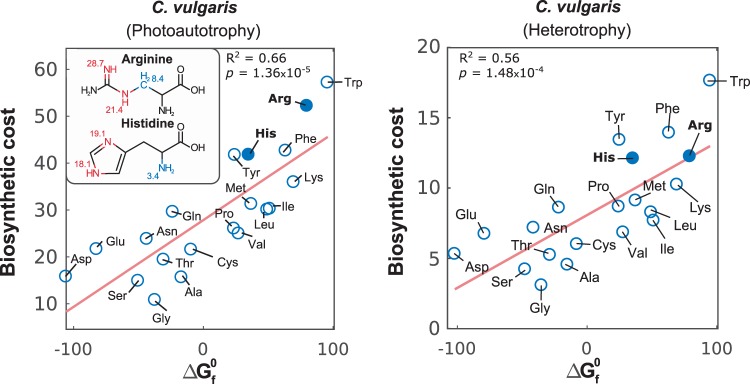
Unraveling interactions among high nitrogen content of histidine and arginine, free energy, and biosynthetic cost. Correlation of calculated biosynthetic costs of amino acids for *C. vulgaris* under phototrophy and heterotrophy and their respective standard Gibbs free energies of formation ($\Delta {\mathrm{G}}_{\mathrm{f}}^0$ in kcal mole^−1^) retrieved from BioCyc^54^. High free energy groups are highlighted in red, low ones in blue, and their contributions according to the Joback’s method^34^ are shown.

This exception to the rule led us to assess the underlying phenomena using the standard Gibbs free energy of formation ($\Delta {\mathrm{G}}_{\mathrm{f}}^0$), which we found to be proportional to biosynthetic cost, as shown in Fig. 4 for *C. vulgaris* (*R*^2^ = 0.66, *p* = 1.35 × 10^−5^ in autotrophy, and *R*^2^ = 0.56, *p* = 1.48 × 10^−4^ in heterotrophy) and Supplementary Fig. 7 for the other organisms. According to Joback’s group contribution method for the estimation of $\Delta {\mathrm{G}}_{\mathrm{f}}^0$^34^, the amino groups contained in the chemical structures of histidine and arginine lie among the most energetic: =NH, >NH (nonring), >NH (ring), and -N= (ring). Since most amino acids contain low free energy -NH_2_ groups, carbon groups tend to have a higher contribution to $\Delta {\mathrm{G}}_{\mathrm{f}}^0$ for all cases except histidine and arginine. In fact, only the groups =C= and >N- (nonring) have a higher contribution than =NH.

## Discussion

Environmental changes associated with nutrient limitation trigger metabolic responses, which have allowed organisms to maintain growth. We study how nitrogen starvation decreases the productivity of essential nitrogen-rich compounds (namely amino acids), causing cells to prioritize higher-energy nitrogen-free biomolecule synthesis, such as those for lipids, carbohydrates, and sterols. Here, we developed a metabolic modeling approach that uses as dynamic constraints time-course metabolomics data to study the effect of biomass composition on flux distributions of five eukaryotic organisms. Simulations predicted the metabolic response of each microorganism to dynamic constraints at organelle and pathway levels. Our calculations showed that nitrogen deprivation caused a major decrease in energy metabolism and the synthesis of nitrogen-rich compounds, as expected.

The mitochondria have been recognized as central hubs of communication among compartments for metabolic and regulation purposes^26^. It has been shown that the cross-talk of the mitochondria with adjacent organelles holds a central role in the signaling for the initiation and completion of apoptosis through the production and transport of signal metabolites, causing an increase of mitochondrial abundance for the synthesis of Bax/Bak proteins^35,36^. However, this change in mitochondrial abundance does not appear to have a basis in metabolic flux demands, since we found that energy metabolism and amino acid precursor synthesis in the mitochondria is expected to decrease in the late stages of culture. Our results showed this reduction is partially due to the high interconnection of the mitochondria through several metabolic functions with the cytosol and the chloroplast (namely the reduction in amino acid synthesis and energy metabolism), that caused the decrease of cytosol and chloroplast activity to affect the mitochondria.

The sensitivity analysis unraveled individual contributions of each biomass precursor to growth of all studied organisms. We determined the biosynthetic cost of all metabolites and showed that the growth was the most sensitive to metabolites with higher biosynthetic cost. Even though we showed that amino acids have some of the lowest biosynthetic costs (Fig. 3), their molar abundance is roughly ten times greater than lipids, resulting in comparable costs of amino acids and lipids. In a deep examination of the biosynthetic costs of amino acids, we found that in reality free energy of compounds was the underlying driver, since our calculations indicated histidine and arginine had higher biosynthetic costs than previously reported^29,30,32,33^. Free energy and molecular weight are not independent, as bigger molecules tend to have higher free energies associated to a larger number of bonds. Rather than discarding molecular weight as a contributor of cost, our results shed light on free energy being the true driver.

The high free energies of arginine and histidine are reflected in the complexity and energy requirement of their biosynthetic pathways. Among the studied organisms, *C. vulgaris, P. tricornutum, Y. lipolytica*, and *S. cerevisiae* are capable to produce arginine and histidine. Intriguingly, differential compartmentalization of arginine biosynthesis does not seem to affect its sensitivity and cost across organisms. On the other hand, flux simulations showed that pathways involved in histidine biosynthesis tend to remain unchanged among phylogenetically distant organisms, in accordance with previous reports^37–39^. Specifically, it is necessary to generate precursors in other pathways, such as the PPP, TCA, acetyl cycles, and nucleotide biosynthetic pathways. In all organisms, arginine biosynthesis consumes ornithine and glutamine, a main nitrogen carrier, which induces an additional cost for the network, considering its high connectivity with the biosynthesis of other amino acids, pigments and nucleotides. Glutamine, specifically, has been identified as one of the most important contributors to carbon and nitrogen metabolism^40^, and a drastic decrease (e.g., 35% decrease in yeast)^6,7^ in its abundance is a common response under nitrogen starvation^2,40,41^. Moreover, the consumption of ornithine fully activates the acetyl cycle, thus adding nitrogen and energy costs by consuming glutamate and ATPs.

For both amino acids, the high free energy of their nitrogen groups translated into the activation of relatively complex pathways, often meaning the consumption of highly connected intermediates and precursors, such as main nitrogen carriers and nucleotide precursors. Arginine requires the full activation of the acetyl cycle, whereas histidine synthesis activates nucleotide synthesis pathways to generate its side chain. In addition, main carriers are principal reactants in the production of both amino acids in all organisms, namely glutamate, glutamine, and ammonium itself. The burden of synthesizing histidine through these pathways is reflected in histidine having some of the lowest abundances in the proteomes of the included organisms (mean abundance = 2.38%, 1st–3rd quartile = 2.32–2.45%), as it has been reported that cells (and even microbial communities) prefer thermodynamic favorability over other efficiency criteria such as cofactor use efficiency^14,33^. Interestingly, arginine and histidine have been associated with metabolic exchange in syntrophic microbial communities^14,42^. The predicted flux exchange may be linked to their high free energy and nitrogen content.

With these results, we demonstrated how nitrogen deprivation shapes the metabolic phenotype in organisms, as well as the underlying phenomena driving these variations. This field has been studied extensively in the past from both experimentation and modeling standpoint^15^; however, the determination of the impact of nitrogen stress at the biochemical reaction-scale had not been addressed. The lack of attention to this phenomenon is mainly caused by the difficulty of quantitative determination. Experimentally, the quantification of intracellular metabolic changes and transport levels is done through microscopy-based methods that have limited quantitative capacity and speed^43^, thus precluding them from analyzing reactome-wide variations. From a modeling perspective, accounting for intracellular transport is still a major challenge, as gene–protein-reaction association data is frequently missing for transporters^1^, thus relegating the addition of transport reactions to gap-filling methods. This issue is only amplified in eukaryotes, where even more information is missing for transporters in organelle membranes. Among other modeling limitations, it is worthy to note that our calculations could be used to reverse-engineer the time-course phenotype adjustment, but could not account for other known responses to nutrient starvation previously identified for phototrophs such as the depletion of pool-like nitrogen reserves^1^ and the breakdown of nitrogen-containing molecules through autophagy^44^ and chlorosis^45,46^. In the case of CHO cells, modeling approaches are not able to account for the increase in mitochondrial number^35,47^, as well as lipid composition variations in close proximity to cellular death^48^.

Here, we showed how condition and time-course composition data can be employed to understand the metabolic processes that an organism undergoes in stress conditions. We analyzed the interplay of metabolites in these processes and identified key drivers in these responses. However, with increased development of novel strategies to obtain more refined omics data, the approach shown in this study can be greatly complemented and can be used to elucidate further time-course pathway coupling and variations that were not captured in the present study due to the aforementioned modeling and data limitations.

## Methods

All simulations were carried out within the MATLAB 2016b (MathWorks Inc.) environment and using the COBRA Toolbox v3.0^49^. FBA was used for flux distribution calculations, and GUROBI 7.5.2 was employed as a solver for the linear optimization problems. Metabolic network visualization was performed using Escher^50^.

### Genome-scale metabolic models

In order to ensure phylogenetic diversity, we included five genome-scale metabolic models under different growth modes: heterotrophy, mixotrophy and/or photoautotrophy, yielding seven organism- and condition-specific simulations. Information regarding genome-scale metabolic model characteristics and growth modes are presented in Table 1. All but one constraint was altered in the models for the analyses in this work: nitrogen exchange. Since we intended to elucidate the change of predicted metabolic requirements without constraint-induced bias, nitrogen exchange bounds were left open. Metabolic models were shared following the standard protocols for computational analysis^51^.

### Formulation of time-course BOFs

Biomass composition data was retrieved for *C. vulgaris*^1^, *P. tricornutum*^4,8–12^, Chinese Hamster Ovary cells^5^, *S. cerevisiae*^6^, and *Y. lipolytica*^7^ (Table 1). In all cases, data was taken before and after nutrient depletion. The timepoints are in hypothetical time units relative to the total duration of culture of each organism, since their phylogenetic diversity render their culture timespan of different scales. The whole culture from nutrient repletion to depletion was divided in 6 stages so that all stages of culture relevant to nutrient depletion were captured: nutrient-replete (1–2), nutrient-deplete (5–6), and a transition stage (3–4). The timespan of culture for each organism is shown in Table 1. At each timepoint, the stoichiometric coefficients of all biomass precursors were calculated from the composition data. In brief, the composition data that was reported in either mass or molar fraction in the datasets was converted into mmol gDW^−1^ h^−1^, as described in standard protocols of metabolic model reconstruction^25,52^ Each model’s production capacity of all measured biomass precursors was first tested, since the metabolites that were measured might not coincide with those initially considered in the network reconstruction. The BOF was constructed including each biomass component one by one, while maintaining the already set values for the catabolic ATP requirement. All metabolites which caused the solution to be infeasible were ignored and the stoichiometric coefficients were then renormalized to add up to 1 g of dry weight (BOFs are provided in Supplementary File 4). In brief, excluded metabolites were carbohydrates which the models of *P. tricornutum* and the yeasts were not able to synthesize, namely d-xylose, d-fucose, mannose, and l-arabinose. Since we wanted to capture the specific effect of the BOF on flux simulations through time, we made sure that the nitrogen was not the limiting factor, so that the shown variations were not due to the mere activation of a constraint. A full summary of the used constraints is provided in Supplementary File 5. Each BOF was used to solve the LP optimization problem at the respective timepoint, employing the loop-less solution method of the function optimizeCbModel available in the COBRA Toolbox. A summary of the constrains is available in Supplementary File 5. The time-course flux distributions that were calculated were used to analyze pathway flux trends and compartment activity variations.

### Hierarchical clustering of pathway flux variation trends

For each timepoint *t*, the absolute difference between the reaction fluxes at *t* and at *t*_0_ was calculated. The variations were normalized by calculating *Z*-scores, which were then processed by the function clustergram, available in Matlab. The distance metric to compute pairwise dissimilarities was Euclidean, and the average linkage was chosen.

### Sensitivity analysis

The sensitivity analysis consisted in the study of how model predictions varied with the composition of each biomass component. The sensitivity of a biomass component was defined as the rate of change of predicted growth rate with a variation of its stoichiometric coefficient. When calculating the sensitivity of metabolite *i*, the composition of all other components in the BOF was randomly varied within their observed range. A total of 100 different hypothetical BOFs were generated for each individual calculation so that the final sensitivity of metabolite *i* was the average of all possible sensitivities within limits that are experimentally possible. For each BOF, the mass fraction of metabolite *i* was varied from the minimum to the maximum observed in the dataset and growth rates were predicted. Finally, the sensitivity *S*_*i*_ of metabolite *i* was estimated through linear regression as the slope of growth rate as a function of the stoichiometric coefficient *p*_*i*_ (Eq. (1)). The intercept *I* was not included in the analysis.

1 $$\mu = \frac{{\partial \mu }}{{\partial p_i}}p_i + I = S_ip_i + I.$$

Model simulations showed that the value of *S*_*i*_ depended on the abundance of other biomass precursors (across all generated BOFs). We calculated the range of variation of *S*_*i*_ as a measurement of the codependence *D*_*i*_ (Eq. (2)).

2 $${\it{D}}_{\it{i}} = {\it{S}}_{{\it{i}},{\rm{max}}} - {\it{S}}_{{\it{i}},{\rm{min}}}.$$

Sensitivity and codependence were correlated with both connectivity and biosynthetic cost. Connectivity was calculated as the number of reactions in which the biomass component appeared with a nonzero stoichiometric coefficient.

### Calculation of biosynthetic cost

For any metabolite *i*, its biosynthetic cost was defined as the amount of ATP moles necessary to produce one mole of *i*. The algorithm to calculate this parameter was taken from Du et al.^33^. The growth rate was first predicted using the existing model. Then, flux through the biomass reaction was set to 95% the predicted value, and the objective function was set to an ATP hydrolysis reaction. A demand reaction was added for each biomass component and a minimal flux of 0.0001 was set for it. The relative decrease in the ADP flux through this reaction after forcing the production of this minimal flux was set as the biosynthetic cost of the metabolite, in units of (mmol ATP) (mmol metabolite)^−1^. The mathematical definition of biosynthetic cost (*A*) for a metabolite *i* is shown in Eq. (3).

3 $${\it{A}}_{\it{i}} = \frac{{\partial {\it{r}}_{{\it{{\rm{ATP}}}}}}}{{\partial {\it{r}}_{\it{i}}}}.$$

### Calculation of compartment activity

Compartment activity was assessed with compartment-specific element (e.g., C, N, O, and P) loads, being carbon and nitrogen loads referred to as activity. Since FBA is a pseudo steady-state method, accumulation as a measurement of load could not be determined. Since all elemental mass that enters a compartment is secreted from it, we estimated carbon and nitrogen loads by the flux of these elements being transported into each compartment by all transport reactions (*R*). Element loads were defined as the molar flux of the element flowing into a certain compartment at a given time, as shown in Eqs. (4) and (5) for the load of element *e* in compartment *c*. This allowed the returned magnitudes to be comparable with each other, and unbiased by reaction stoichiometry.

4 $${\it{l}}_{{\it{i}},{\it{c}}}^{\it{e}} = \mathop {\sum}\limits_{{\it{r}} = 1}^{\it{R}} {{\it{v}}_{\it{r}}} \ast {\it{\gamma }}_{{\it{i}},{\it{r}}} \ast {\it{N}}_{\it{i}}^{\it{e}},$$

5 $${\it{L}}_{\it{c}}^{\it{e}} = \mathop {\sum}\limits_{{\it{i}} = 1}^{\it{M}} {{\it{l}}_{{\it{i}},{\it{c}}}^{\it{e}}},$$

where $l_{i,c}^e$ is the amount of element *e* carried by metabolite *i* into compartment *c*. ${\mathrm{v}}_{\mathrm{r}}$, ${\mathrm{\gamma }}_{{\mathrm{i}},{\mathrm{r}}}$, and ${\mathrm{N}}_{\mathrm{i}}^e$ are the flux of reaction *r*, the stoichiometric coefficient of *i* in it, and the number of *e* atoms in *i*'s molecular structure, respectively. Only positive ${\mathrm{\gamma }}_{{\mathrm{i}},{\mathrm{r}}}$ were included, since the mass balance is bound to close because of the assumption of pseudo-steady state. Finally, ${\mathrm{L}}_c^e$ is the load of *e* in *c*.

### Determination of upregulation and downregulation of reactions

To assess significant upregulation and downregulation of reactions, we sampled the solution space for each model and timepoint. Sampling was carried out using the gpSampler function of the COBRA Toolbox^23^ in close proximity to the optimal solution space by constraining the LP problem to a minimum of 90% the optimal solution^53^, with a sample size of 5000 points for each timepoint. Reactions were considered to be upregulation or downregulated if the paired one-tailed (right and left respectively) Student’s *t* test rejected the null hypothesis with a *p* value lower than a significance level of 0.05, with reaction flux distributions at the initial and final time points as inputs.

## Supplementary information

Supplemental Material

Dataset 1

Dataset 2

Dataset 3

Dataset 4

Dataset 5

## Data Availability

All relevant data is contained in this document and the supplementary files. MATLAB scripts and functions are available in the *sensitivityAnalysis* repository at https://github.com/jdtibochab/sensitivityAnalysis.
