# Supplementary material for: Dynamic resource allocation drives growth under nitrogen starvation in eukaryotes
Source: NPJ Syst Biol Appl. 2020 May 15;6:14. doi: 10.1038/s41540-020-0135-y (PMC7229059; doi:10.1038/s41540-020-0135-y)
Supplement: Supplementary file 1 — Supplemental Material [file 41540_2020_135_MOESM1_ESM.pdf]

Supplementary Information

Dynamic resource allocation drives growth under nitrogen starvation in eukaryotes

Juan D. Tibocha-Bonilla[1,2,3], Manish Kumar[2], Anne Richelle[2], Rubén D. Godoy-Silva[3], Karsten Zengler[2,4,5], Cristal Zuñiga[2]*

[1]Department of Bioinformatics and Systems Biology, University of California, San Diego, 9500 Gilman Drive, La Jolla, CA 92093-0760, USA

[3]Department of Pediatrics, University of California, San Diego, 9500 Gilman Drive, La Jolla, CA 92093-0760, USA

[3]Grupo de Investigación en Procesos Químicos y Bioquímicos, Departamento de Ingeniería Química y Ambiental, Universidad Nacional de Colombia, Bogotá, D.C., Colombia

[4]Department of Bioengineering, University of California, San Diego, La Jolla CA 92093-0412, USA

[5]Center for Microbiome Innovation, University of California, San Diego, 9500 Gilman Drive, La Jolla, CA 92093-0403, USA

*Corresponding author: Cristal Zuñiga

This file contains:

Captions of Supplementary files 1 to 5
Supplementary figures

Content

Captions of Supplementary files 3
Supplementary Figures 4
Supplementary Figure 1.Average time-course metabolomics data for all five organisms. 4
Supplementary Figure 2.Correlation of sensitivity with biosynthetic cost and connectivity. 5
Supplementary Figure 3. Correlation of codependence with biosynthetic cost and connectivity. 6
Supplementary Figure 4. Nitrogen load for compartments available in each model through the time of culture. 7
Supplementary Figure 5. Correlation of amino acid biosynthetic cost with N/C ratio. 8
Supplementary Figure 6. Correlation of biosynthetic cost with standard Gibbs free energy of formation retrieved from BioCyc. 9

Captions of Supplementary files

Supplementary File 1. Up- and down-regulation results for all reactions in the models.
Supplementary File 2. Fraction of up- and downregulated reactions in each subsystem.
Supplementary File 3. Correlation coefficients of biosynthetic costs based on NADH, NADPH and ATP.
Supplementary File 4. Biomass Objective Function stoichiometric coefficients in all timepoints.
Supplementary File 5. Summary of constraints used for flux distributions.

Supplementary Figures

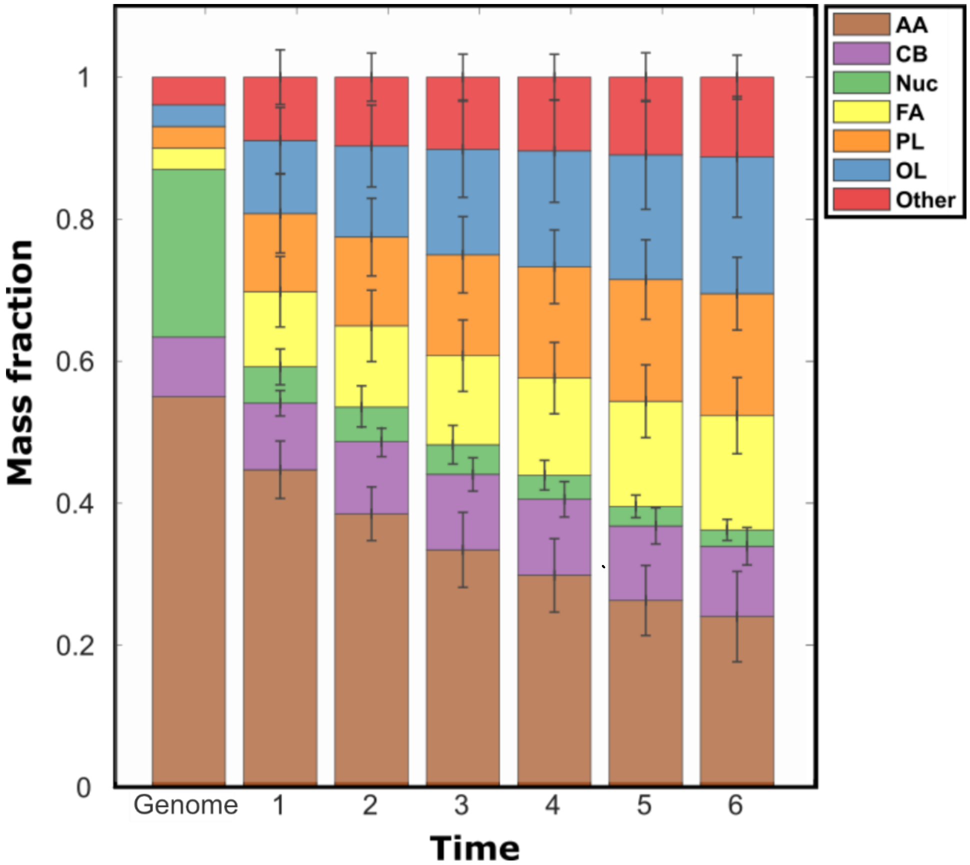

Supplementary Figure 1. **Average time-course metabolomics data for all five organisms.** Biomass composition data was collected from previous reports of experimental metabolomics measurements along the timespan of culture from early stage (timepoints 1-3: nutrient-sufficient) to the late stage (timepoints 4-6: nutrient stress), and standard deviation is represented by the error bars. Biomass components are abbreviated as follows: amino acids (AA), carbohydrates (CB), nucleotides (Nuc), fatty acids in the form of acylglycerols (FA), phospholipids (PL) and other lipids (OL). Calculated biomass composition (first bar) based on the genome sequence, composition was determined following the well stablished protocol for reconstruction of genome-scale metabolic models (Thiele and Palsson, 2010).

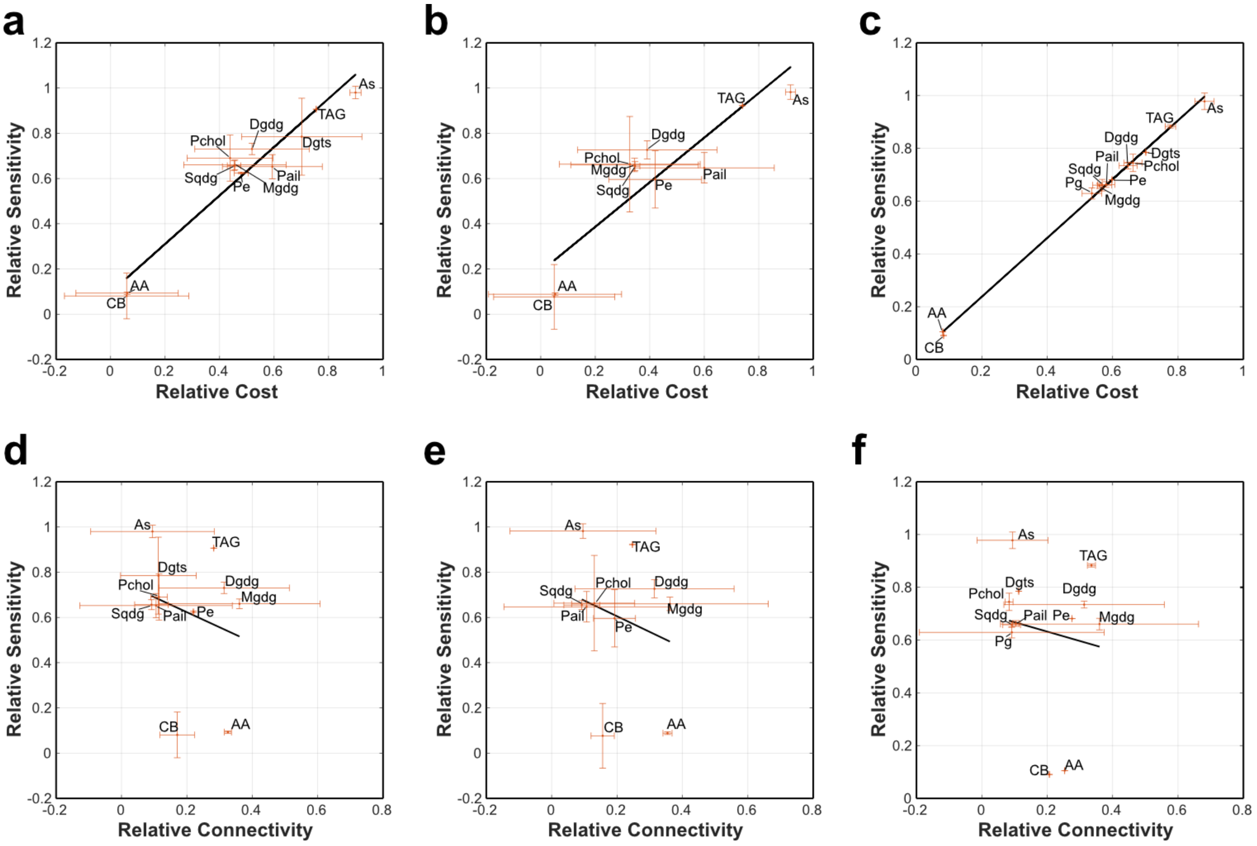

Supplementary Figure 2. Correlation of sensitivity with biosynthetic cost and connectivity. Sensitivity was correlated with biosynthetic cost (A – C) and connectivity (D – F) for all organisms (A and D), heterotrophs (B and E) and phototrophs (C and F), and standard deviation is represented by the error bars. While there is a clear proportional trend between sensitivity and cost, connectivity appears not to significantly affect the sensitivity of metabolites.

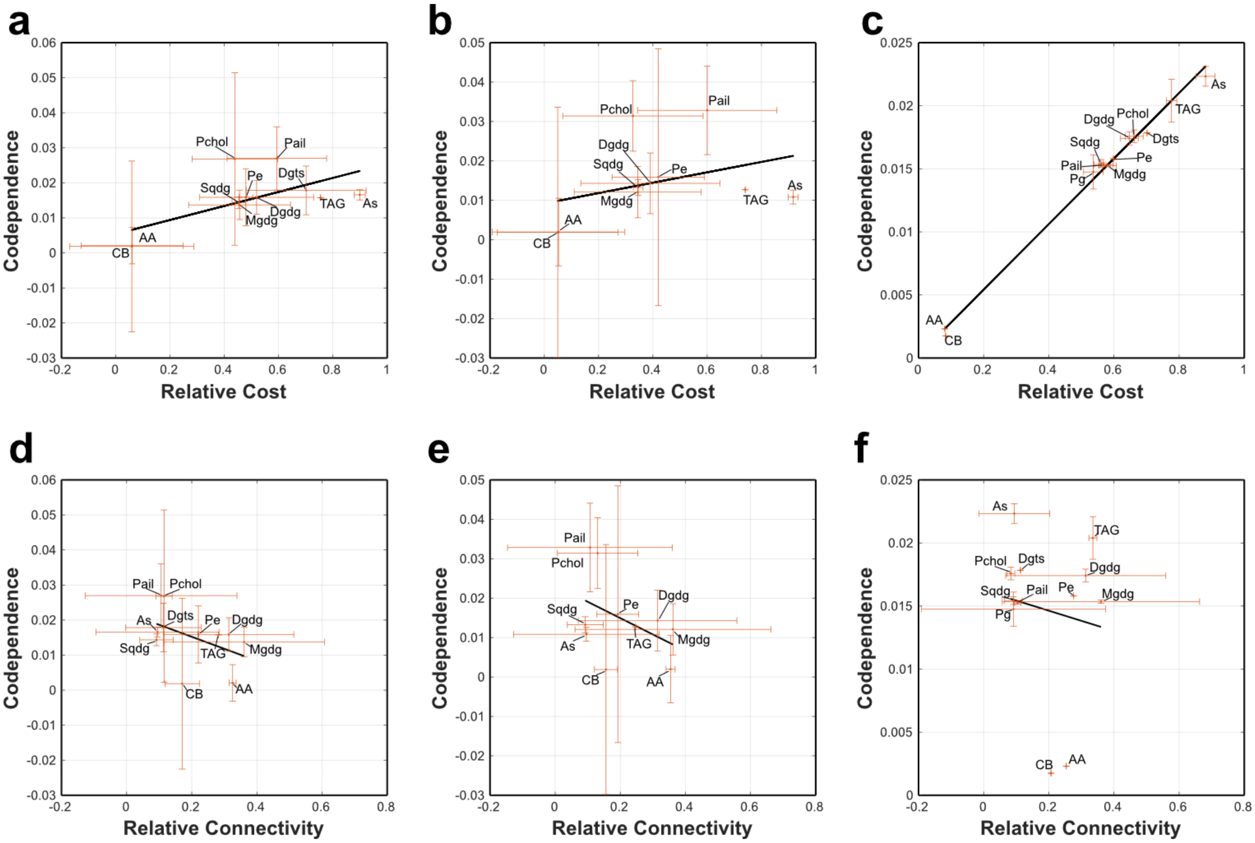

Supplementary Figure 3. Correlation of codependence with biosynthetic cost and connectivity. Codependence was correlated with biosynthetic cost (A – C) and connectivity (D – F) for all organisms (A and D), heterotrophs (B and E) and phototrophs (C and F), and standard deviation is represented by the error bars. While there is a clear proportional trend between codependence and cost, connectivity appears not to significantly affect the codependence of metabolites.

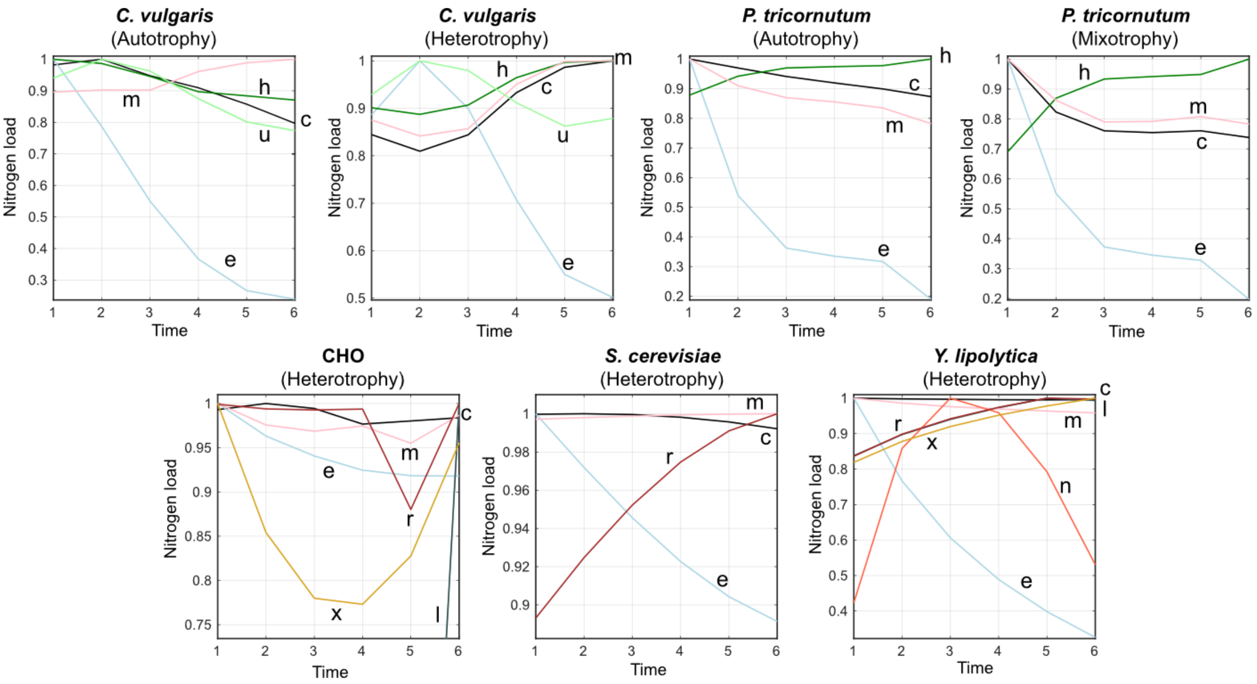

Supplementary Figure 4. Nitrogen load for compartments available in each model through the time of culture. *C. vulgaris* (autotrophy and heterotrophy), *P. tricornutum* (autotrophy and mixotrophy), CHO, *S. cerevisiae*, *Y. lipolytica*. Included compartments are: mitrochondria (m), chloroplast (h), extracellular environment (e), thylakoid lumen (u), cytoplasm (c), glyoxysome (x), endoplasmic reticulum (r), lysosome (l), and nucleus (n).

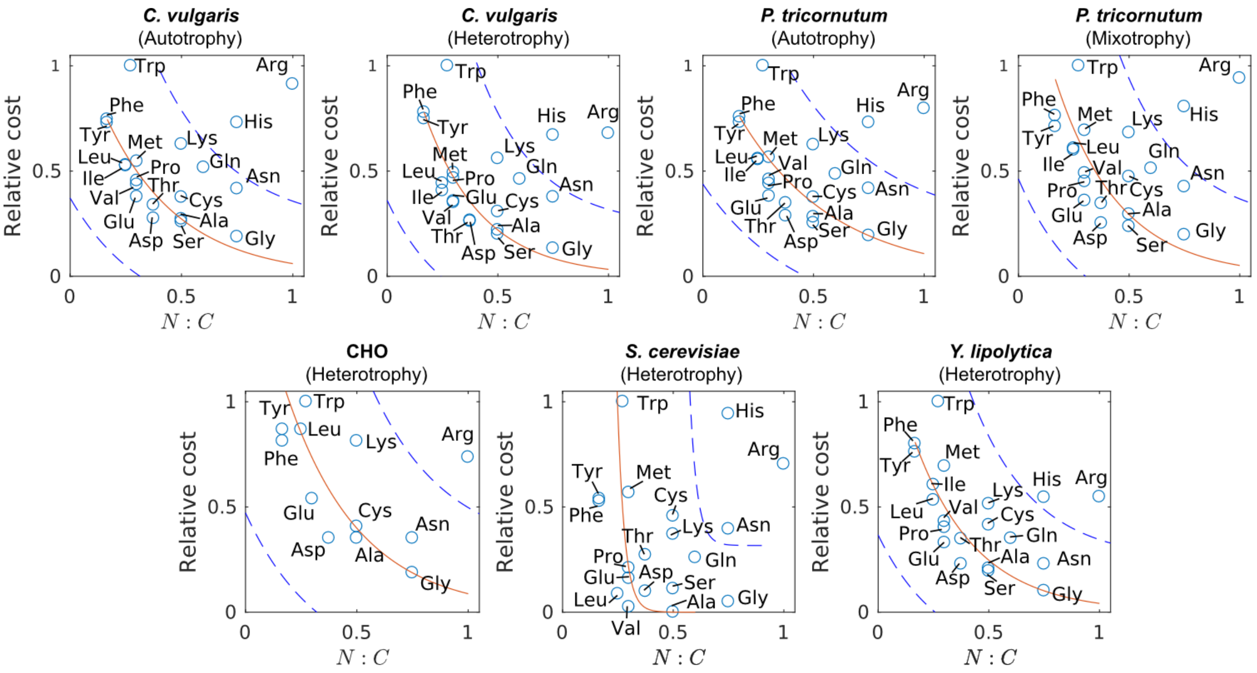

Supplementary Figure 5. **Correlation of amino acid biosynthetic cost with N/C ratio.** In all organisms, the biosynthetic cost of amino acids appears to be mainly driven by the molecular weight, as lower N/C ratios correspond to higher biosynthetic costs. However, for the nitrogen-rich histidine and arginine, nitrogen appears to have a much greater contribution.

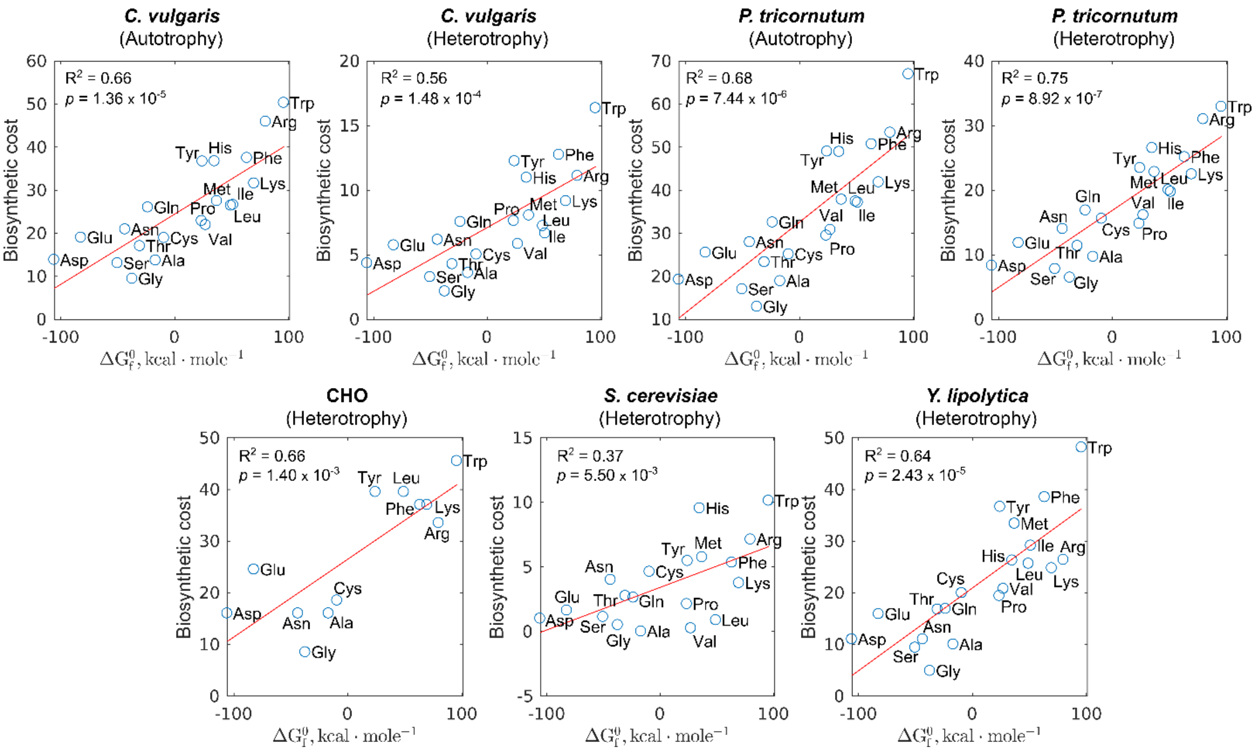

Supplementary Figure 6. Correlation of biosynthetic cost with standard Gibbs free energy of formation retrieved from BioCyc. Biosynthetic cost was shown to be mainly driven by the energy level of amino acids, represented here as the Gibbs free energy of formation, as opposed to previous reports claiming that the main driver was solely the molecular weight.
